# A pragmatic randomised controlled trial of SAFMEDS to produce fluency in interpretation of electrocardiograms

**DOI:** 10.1186/s12909-020-02021-8

**Published:** 2020-03-31

**Authors:** Louise Rabbitt, Dara Byrne, Paul O’Connor, Miroslawa Gorecka, Alan Jacobsen, Sinéad Lydon

**Affiliations:** 1 School of Medicine, National University of Ireland Galway, 1 Distillery Road, Galway, Ireland; 2 Irish Centre for Applied Patient Safety and Simulation, National University of Ireland Galway, Galway, Ireland; 3 School of Medicine, Department of General Practice, National University of Ireland Galway, Galway, Ireland; 4 St James’s Hospital, James’s St North, Ushers Co., Dublin, Ireland; 5 Osler Medical Residency, Johns Hopkins Hospital, 1800 Orleans St, Baltimore, MD 21287 USA

**Keywords:** Medical education, Electrocardiogram, Behaviourism, Behavioural fluency, SAFMEDS

## Abstract

**Background:**

SAFMEDS (Say-All-Fast-Minute-Every-Day-Shuffled) is a flashcard-type behavioural instructional methodology, involving one-minute learning trials that function both as practice and assessment, used to facilitate the development of fluency in a behaviour.

The primary research question was whether SAFMEDS engenders improvement in performance beyond that conferred by usual teaching. A secondary research question was whether SAFMEDS is an effective method of producing fluency in Electrocardiogram (ECG) interpretation.

**Methods:**

A pilot study was conducted to determine sample size required to power the pragmatic randomised controlled trial (RCT). For the subsequent RCT, participants were randomly assigned to a “usual teaching” control group (*n* = 14) or the SAFMEDS intervention group (*n* = 13), with the recognition of 15 cardiac conditions on ECGs (e.g., atrial fibrillation, complete heart block) targeted. Intervention group participants’ performance was tracked over eight weeks as they worked towards achieving the fluency criterion. Percentage accuracy in ECG interpretation was assessed at baseline and post-test for both groups. An ANCOVA was conducted to assess for differences in the performance of the intervention and control group at post-test while controlling for the baseline performance of participants. At post-test, the numbers of participants achieving fluency within the intervention group was examined.

**Results:**

A large effect size of SAFMEDS (partial η^2^ = .67) was identified when controlling for the effects of baseline performance. At post-test, the intervention group significantly outperformed (*M* = 61.5%; *SD* = 12.1%) the control group (*M* = 31.6%; *SD* = 12.5%, *p* < .001). In total, 7 of 13 intervention group participants achieved fluency. Participants required an average of 51.9 one-minute trials (*SD* = 18.8) to achieve fluency.

**Conclusions:**

SAFMEDS offers a useful adjunct to usual teaching within medical education. Further research could assess whether learning retains, is stable, and transfers to clinical practice.

## Background

Behavioural fluency is defined as “the combination of accuracy plus speed in responding that comprises competent performance” and has emerged as a concept within behaviourism [1]. In practice, fluency looks like automaticity in behaviour, correct responding without hesitation, or effortless performance [1]. Behavioural fluency differs from traditional concepts of mastery (commonly used in simulation-based education), as behavioural fluency focuses not only on the accuracy or correctness of performance, but also its pace [2]. Behaviours taught to fluency have been shown to maintain better over time (*Retention*), to transfer to other contexts (*Generalisation*) and to endure despite distraction [1, 3, 4]. Interventions targeting behaviour fluency: 1) are typically criterion-referenced, meaning that learners work towards achieving a pre-determined ‘expert’ standard that is time-based [5]; 2) are focused on providing learners with opportunities to practice the behaviour, a key element of the learning process often absent in educational programs [6]; and 3) involve both continuous measurement of behaviour and performance feedback for the learner [5, 6]. One interventional strategy used to produce fluency is the Say-All-Fast-Minute-Every-Day-Shuffled (SAFMEDS) methodology.

SAFMEDS is a flashcard-type behavioural instructional methodology used to assist learners in developing fluency in a behaviour using one-minute timed learning trials that function both as practice and assessment [7, 8]. Prior to a one-minute SAFMEDS trial, a learner shuffles their pack of SAFMEDS cards. During the trial, the learner repeatedly responds aloud verbally to a stimulus, which may be visual or textual, printed on the front of each SAFMEDS card, then checks the correspondence of their answer to the correct answer shown in text on the back of the card before placing the card into a “correct” or “incorrect” pile as appropriate. The learner proceeds through as many cards as possible during the one-minute trial. Following the trial, the learner reviews the cards, and associated content, they identified incorrectly. SAFMEDS is a learner-centric instructional method and allows the learner to respond at their own pace and immediately receive corrective feedback [7]. SAFMEDS has been used effectively in a number of educational settings with a variety of different learners and many different target behaviours [9, 10]. Among medical students, SAFMEDS has been shown to produce significantly better outcomes in dermatology diagnostic skills than usual teaching only [2].

This study will describe the application of SAFMEDS to the teaching of electrocardiogram (ECG) interpretation. Deficiencies in the interpretation of ECG abnormalities, including critical life-threatening abnormalities, have been identified in several studies of undergraduate [11] and postgraduate learners [12–14]. ECG interpretation is taught in medical school through a variety of methods [15–17], but there is no agreement on the most effective approach [18].

The primary research question of this pragmatic randomised controlled trial (RCT) [19] of the application of SAFMEDS to teaching ECG interpretation was: does SAFMEDS engender any improvement in performance beyond that conferred by usual teaching? A secondary research question was: is SAFMEDS an effective method of producing behavioural fluency in ECG interpretation?

## Methods

### Research design

This study used a pragmatic RCT design, meaning that the experiment was conducted in a real-life educational setting (reflecting everyday practice) rather than within artificial, tightly controlled conditions [20]. The participants randomly assigned to the intervention group received a brief introductory teaching session on ECG interpretation, the SAFMEDS intervention, and the medical school’s usual teaching on ECG interpretation, which included clinical rotations through cardiology and a 2-h small-group tutorial on ECG interpretation from a consultant cardiologist. The participants assigned to the control group received the brief introductory teaching session and the medical school’s usual teaching.

### Outcome measures

The primary outcome measure in this study was percentage accuracy, as measured for both experimental groups at baseline and post-test. SAFMEDS is predicated on the concept of behavioural fluency. Behavioural fluency requires both accuracy and pace. There is precedent to using percentage accuracy in evaluations of SAFMEDS using between-groups experimental designs [2, 21, 22]. The secondary outcome was attainment of behavioural fluency. This secondary outcome was assessed through the consideration of the numbers of participants in the intervention group who achieved behavioural fluency (i.e., met the pre-specified fluency criterion; described below).

### Pilot study

A pilot for this study was conducted in early 2017 to estimate the effect size of the SAFMEDS intervention and to determine the number of participants needed to power a subsequent RCT. Pilot data from 18 medical students, who engaged with a brief introductory teaching session on ECG interpretation and the SAFMEDS intervention (with an average of 10.3 one-minute SAFMEDS trials completed by participants), showed that the percentage correct on an ECG test (test format and development described in detail in ‘materials’ subsection that follows) increased from a mean of 15.8% (*SD* = 7.3%) at baseline to 47.8% (*SD* = 14.1%). The data are indicative of a large effect size of the intervention (Cohen’s *d* = 2.05). We used this information within G*power [23, 24] to compute the sample size required. This analysis indicated a total sample size of twelve (six per group) would be required. Additional participants were recruited beyond this for the RCT in order to account for the possibility of attrition and ensure that our sample size was in line with other published studies.

### Setting, recruitment and participants

This study was conducted on-site at one medical school. In September 2017, final-year medical students (*n* = 104) from a 5-year undergraduate program in an Irish university were invited to participate in this study by LR via a post on their virtual learning environment that provided an overview of the study and asked them to attend a voluntary information session if they were interested in participating.

### Ethical approval

Ethical approval was received from the National University of Ireland Galway’s Research Ethics Committee (ref: 17-Jan-10).

### Target behaviour

The target behaviour was the correct identification of the cardiac condition or normal cardiac activity in an ECG. For the purpose of this study, senior cardiologists identified 15 cardiac conditions that they believed to be important for a qualified doctor to be able to diagnose rapidly (see Table 1).

**Table 1:** Cardiac conditions targeted within the study and number of related cards within each SAFMEDS pack

Diagnoses	Number of cards with each pack
Normal	12
First-degree AV block	3
Anterior STEMI	3
Atrial Flutter	3
Atrial Fibrillation	3
Complete Heart Block	3
Inferior STEMI	3
Lateral STEMI	3
Left bundle branch block (LBBB)	3
Mobitz I	3
Paced	3
Posterior STEMI	3
Right bundle branch block (RBBB)	3
Trifascicular block	3
Wellens syndrome	3
Wolff-Parkinson-White Syndrome	2

### Materials

The materials consisted of training materials (in the form of a set of SAFMEDS cards), two 35-item tests (used for baseline and post-testing) and data recording sheets.

It was necessary to collect multiple different exemplar ECG images for each cardiac condition, and representing normal cardiac activity, in order to produce the SAFMEDS cards and the tests. The ECG exemplars used were selected from open-access repositories, and the researchers’ personal collections. All images were reviewed by three physicians with expertise in cardiology, and were included if all agreed that the images constituted good exemplars of the targeted conditions.

The SAFMEDS card pack was developed using the ECG exemplars collected. Each SAFMEDS card was 15 cm × 10.5 cm and showed a 12-lead ECG on one side and specified the correct diagnosis in text on the other side. Each participant received a set of 56 cards (21.4% of cards showing normal cardiac functioning), the composition of which is shown in Table 1, and some simple data recording sheets that allowed for the recording of the date of each trial along with the number of corrects and incorrects observed.

Two 35-item ECG tests (forms A and B) were also compiled using the remaining exemplar ECG images that had been collected. These tests instructed participants to identify the cardiac condition represented in each ECG. Each test page presented two ECGs with a space beneath each image for the participant to write the cardiac condition identified in the image presented. Pilot testing was conducted with four physicians with varying levels of expertise relating to ECG interpretation to ensure equivalency in difficulty between the two test forms. Each test included two examples of each of the 15 targeted cardiac conditions and five ECG images showing normal cardiac activity (14.3%), with these images presented in random order.

### Procedures

#### Preparation

A fluency criterion represents the standard at which an expert, or person fluent in the target behaviour, would be expected to perform. For the purpose of this study, a senior cardiac physiologist was selected as the expert. The rationale for the choice was that the core job of a senior cardiac physiologist is the conduct and interpretation of ECGs. The fluency criterion was established by asking one senior cardiac physiologist to complete three one-minute SAFMEDS trials: the median score was taken from this expert’s scores. It was therefore required that, in order to be deemed as “fluent”, participants correctly identify 17 cardiac conditions in ECG images presented on the SAFMEDS cards within two successive one-minute trials.

#### SAFMEDS evaluation

**Fig. 1:**
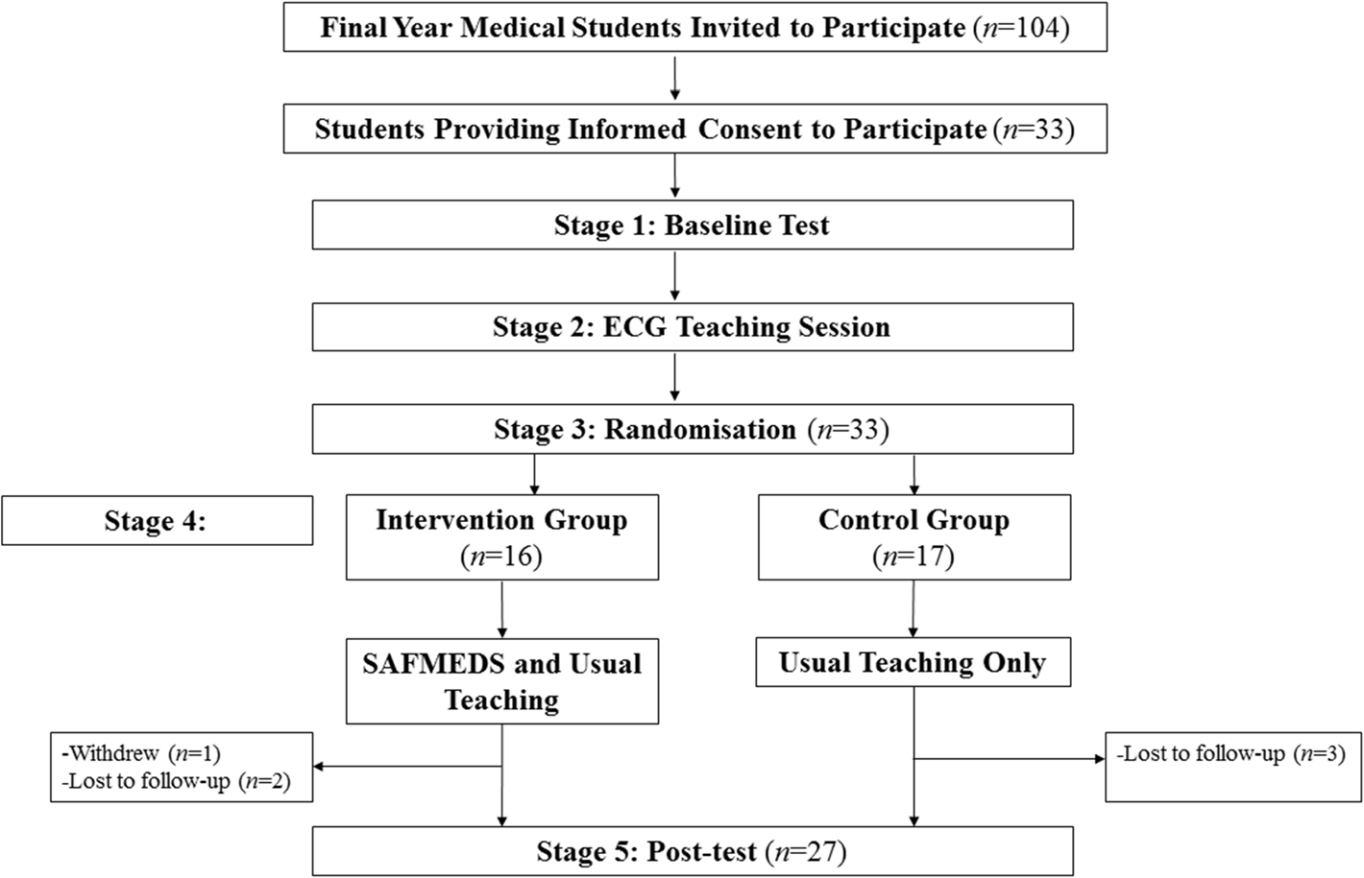
Flowchart depicting the research activities of the intervention and control group across the study

A summary of study procedures is shown in Fig. 1. Each of the five stages of the intervention are outlined in detail below.

#### Stage 1. Baseline test (all participants; September 2017)

Participants were given 25 min to complete a 35-item test. The two forms of the test were distributed randomly during the baseline testing session, with approximately half of the participants receiving each test form.

#### Stage 2. ECG teaching session (all participants; September 2017)

Baseline testing was followed by a 40-min ECG teaching session for all participants. The teaching was delivered by a senior cardiology doctor who emphasised recognising key patterns within ECGs, with a particular focus on the 15 targeted cardiac conditions. This tutorial was recorded, and the recording and associated presentation slides were made available to all participants by email.

#### Stage 3. Randomisation (all participants; September 2017)

Simple randomisation was subsequently completed by LR and SL by asking participants to pull pieces of paper marked either ‘control’ or ‘intervention’ from a hat [25].

#### Stage 4. SAFMEDS and usual teaching (intervention group; September–November 2017) or usual teaching only (control group; September–November 2017)

Participants assigned to the intervention group were each given a set of SAFMEDS cards and a brief tutorial on how to use them. For each SAFMEDS trial, participants were instructed to shuffle their set of cards, set a one-minute timer and then examine each card in turn, say aloud the cardiac condition they thought best fit the ECG pattern presented, or alternatively if they identified the patterns as normal cardiac activity, and check their answer against the correct answer on the reverse of the card. Participants then placed the card in a ‘correct’ or ‘incorrect’ pile as appropriate. At the end of each trial, participants recorded their number of corrects and incorrects on a data recording sheet and reviewed cards identified incorrectly. Participants were encouraged to complete at least one trial daily, but the frequency of trials was ultimately at participants’ discretion.

Intervention group participants emailed the researchers a copy of their data recording sheet weekly. Using this data, an online platform, Chartlytics©, was used to generate a standard celeration chart (SCC; a semi-logarithmic chart that depicts the frequency of a target behaviour and allows for the ascertainment of whether a growth in learning is occurring or whether a change in instructional tactics is required) for each participant [26]. Participants received an updated copy of their chart weekly with written feedback. If a learner’s data indicated that they were not progressing (i.e., corrects not increasing, incorrects increasing) they were encouraged to make note of the conditions they were incorrectly identifying and to revise the content of the recording and/or presentation slides associated with these.

Both the intervention and control group continued to receive access to usual teaching in the medical school, including its offerings relating to cardiology and ECG interpretation.

#### Stage 5. Post-test (all participants; November 2017)

At post-test, participants completed the form of the test that they had not completed at baseline. Intervention group participants who achieved fluency completed the post-intervention test at this time. Intervention group participants who did not achieve fluency, and control group participants, completed the post-test eight weeks after baseline testing. The eight-week cut-off was logistical and chosen to ensure that there was no interference with students’ exam preparations or exams.

### Data analysis

#### Does SAFMEDS engender any improvement in performance beyond that conferred by usual teaching?

The performance of the intervention and control group was compared using an ANCOVA. Within this ANCOVA, the independent variable was group assignment (with two levels, either intervention group or control group). The dependent/outcome variable was percentage accuracy as measured at the post-test timepoint. Percentage accuracy at baseline was entered as a covariate in order to control for participants’ abilities at the beginning of the study and to examine the effects of the intervention while controlling for this variable. Partial η^2^ is a widely used measure of effect size in educational research [27] and was calculated in the current study as an indication of the effect size associated with the SAFMEDS intervention, beyond usual teaching.

#### Is SAFMEDS an effective method of producing behavioural fluency in ECG interpretation?

This research question was addressed by considering the proportions (*n;* %) of participants in the intervention group who achieved fluency during the eight-week intervention phase.

## Results

### Participants

A total of 33 final-year medical students provided informed consent to participate. Of these, 16 were randomly assigned to the intervention group and 17 to the control group. Among the intervention group participants, one participant withdrew (citing a high workload as the reason) and two others were lost to follow-up; their data were removed from subsequent analysis and are not reported. This resulted in an intervention group comprising of 13 participants (9 women, 4 men) with a mean age of 23.2 years (*SD* = 1.2). Three control group participants were lost to follow-up and their data were excluded. The final control group included 14 participants (9 women, 5 men) with a mean age of 23.6 years (*SD* = 1.6). There was no significant difference in mean age (*t*(25) = .6, *p* = .54) or gender distribution across the groups (*χ*^2^ = .07, *p* = .56).

### Does SAFMEDS engender any improvement in performance beyond that conferred by usual teaching?

**Fig. 2:**
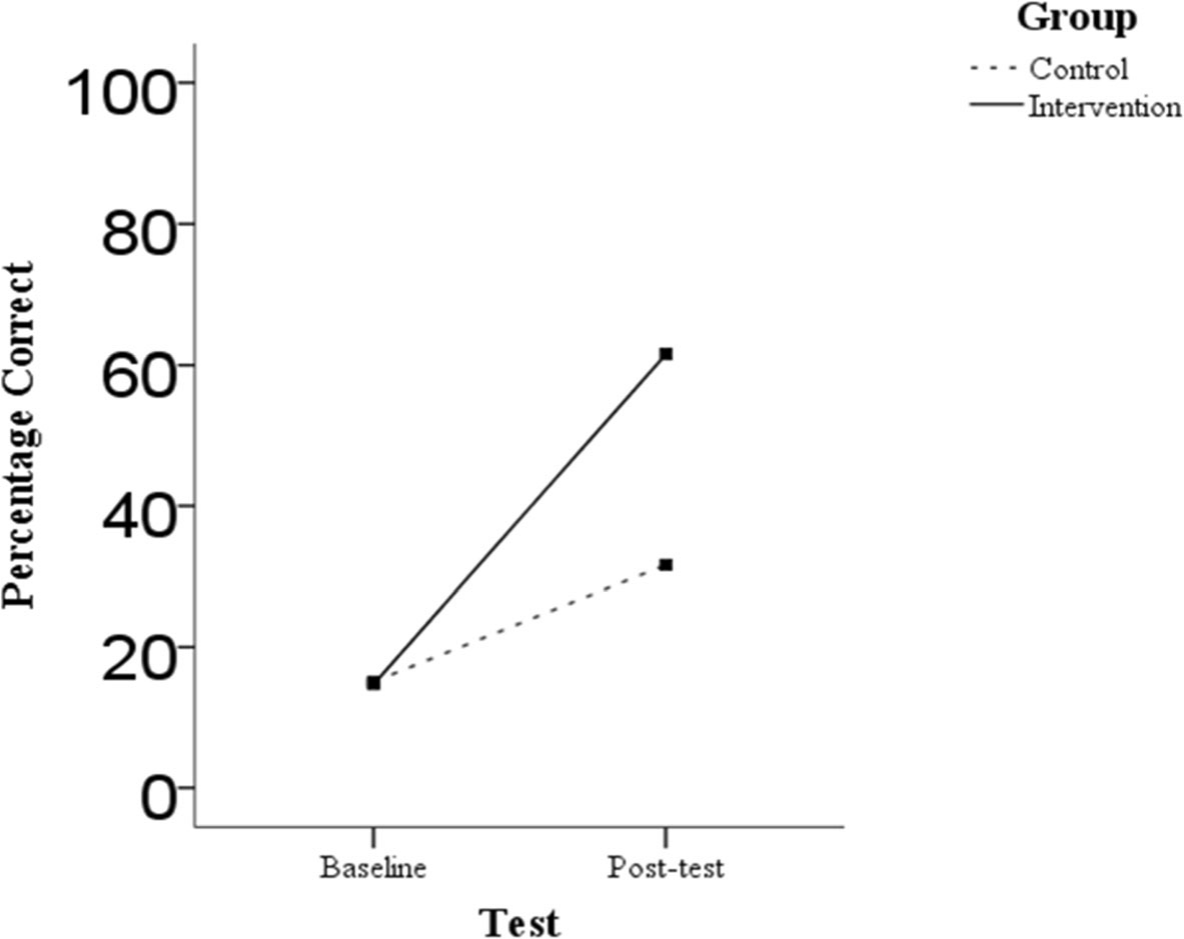
Line graph depicting intervention group performance as compared to control group at baseline and post-test. Note: At baseline, the groups performed comparably. Percentage correct was 14.7 (SD=18.1) in the intervention group and 15.1 (SD=9.9) in the control group

Figure 2 offers a graphical presentation of the performance of the intervention and control group, as indicated by percentage accuracy, at baseline, and post-test. An ANCOVA was conducted to compare the performance of the intervention and control groups at the post-test timepoint while controlling for participants’ baseline performance. The covariate, percentage accuracy at baseline, was significantly related to post-test performance, *F*(1,24) = 6.21, *p* = .02. There was also a significant effect of group assignment identified on post-test performance, *F*(1,24) = 48.8, *p* < .001, partial η^2^ = .67 (large effect). As can be seen in Fig. 2, while both groups improved across the duration of the study, the degree of improvement observed in the intervention group was significantly greater than that in the control group. At post-test, the intervention group had a mean score of 61.5% (*SD* = 12.10) while the control group had a mean score of 31.63% (*SD* = 12.5).

### Is SAFMEDS an effective method of producing behavioural fluency in ECG interpretation?

A total of seven participants (of 13; 53.8%) met the criterion for fluency. These participants required an average of 51.9 trials (*SD* = 18.8) to achieve fluency. The remaining six participants completed a mean of 31.5 learning trials (*SD* = 28.6).

## Discussion

The aim of this pragmatic RCT was to determine whether SAFMEDS, a behavioural instructional methodology, could lead to fluency in ECG interpretation among final year medical students, and to establish whether SAFMEDS engendered any improvement in performance beyond that conferred by usual teaching.

The usefulness of SAFMEDS as an adjunct to usual teaching in medical education is apparent. A large effect size of the intervention was discerned with an average of only 42.5 min of practice time across the intervention group. Our findings are consistent with other studies in medical education showing the efficacy of fluency-based instructional methods [2, 3] and the effects of SAFMEDS observed in other educational domains [2, 9, 10, 21, 28]. The positive impact of SAFMEDS may not be entirely surprising given the well-established impact of test-enhanced learning in medical education [29] and the dearth of opportunities for practice, or examples, offered by many educational programs [6]. ‘Time-on-task’ is an important confounder within this study, as the intervention group are likely to have devoted 30–60 min to improving their performance in interpreting ECGs over the eight weeks which the control group may not have done. However, SAFMEDS nonetheless offers a useful means of structuring and encouraging this ‘time-on-task’ for students. Therefore, SAFMEDS may yield greater gains than other forms of ‘time-on-task’ outside of the classroom that students may have undertaken. Future research which examines this issue would be of much use. Beyond this, future research exploring the efficacy of SAFMEDS for improving other important target behaviours within medical education would contribute substantially to our understanding of SAFMEDS’ efficacy, and its potential as an adjunct teaching method in health professions’ curricula.

In the current study, only 7 of 13 participants reached fluency as a result of using the SAFMEDS intervention. Other evaluations of SAFMEDS have also noted that not all participants achieve the fluency criterion although substantive improvements are observed [21]. It is perhaps unsurprising that participants who achieved fluency completed a higher average number of trials (*M* = 51.9) than participants who did not achieve fluency (*M* = 31.5). Future research focused specifically on examining individual characteristics that predict optimal use of the SAFMEDS intervention, the duration required to achieve fluency, higher test scores, and better retention of learning would greatly advance our knowledge of the efficacy of SAFMEDS and how best it can be employed in health sciences education. Further, our data suggest that it will be important for researchers, or those employing SAFMEDS in real-world contexts, to give consideration to the strategies or supports that may be employed to: 1) motivate students to engage in SAFMEDS practice, and 2) support students who are not progressing or who fail to achieve fluency. The elucidation of such strategies is essential to ensure that all can succeed and will develop the core clinical skills targeted.

An alternate explanation for participant’s failure to achieve fluency is that the fluency criterion may have been set too high. This issue may relate to the choice of a senior cardiac physiologist as the reference for the criterion-setting, rather than a cardiology doctor, which may be critiqued. However, there has been previous discussion about outcomes-based education and whether we “disempower learners and set adequacy rather than excellence as the goal of medical curricula” [28]. Setting a real-world standard by which to judge trainees is a complex task. Setting an exigent fluency criterion may encourage learners to achieve a higher level of performance than a lower standard would. Guadagnoli and Lee [30] have previously suggested that there exists an ‘optimal challenge point’, whereby task difficulty and learner ability interact to result in an appropriate level of challenge. They have suggested that the optimal challenge point varies depending on these variables and across a training programme as learner performance improves. The fluency, or expert, criterion is key to SAFMEDS and other interventions targeting behavioural fluency and it is typical to establish just one standard that is determined to be indicative of behavioural fluency, and which considers both accuracy and pace of expected performance [5]. However, future research should give consideration to fluency criterion/standard setting within such interventions. This research may benefit from a consideration of the establishment of the fluency criterion through the testing of multiple experts, examining the effects of SAFMEDS with and without a specified fluency criterion, and considering whether varying the criterion by participants and/or in response to progression of learning ultimately impacts achievement and the retention of learning.

### Limitations

A number of limitations must be noted. First, there is substantive, and well-founded, debate surrounding the use of RCTs in education research [31, 32], and whether there is truly value in comparing the impact of an educational intervention to no intervention or ‘teaching as usual’ when we might always expect some teaching or extra teaching to improve performance. This study, which compared the SAFMEDS intervention to ‘teaching as usual’, is perhaps an exemplar of this issue. It is well recognised that medical education can be improved beyond standard teaching practices and, for this reason, it might be considered unsurprising that the intervention group participants who utilised SAFMEDS outperformed their peers in the control group. The issue of an underdeveloped comparison is also reflected in our sample size calculation which suggested that only six participants were required in each group. However, in spite of these limitations, we suggest there is nonetheless value in our endeavour which has ascertained the effect size that can be achieved with a short duration of SAFMEDS practice (< 60 min) and which introduces SAFMEDS as a useful adjunct to teaching as usual to a medical education audience. Future research on SAFMEDS in medical education may benefit from employing alternative research designs and considering the acceptability or feasibility of using SAFMEDS within a curriculum or comparing its effects to those of other novel forms of teaching in medical curriculum. Second, SAFMEDS does not provide meaningful clinical context for the ECGs used [18, 33]. This demonstrates a lack of ecological validity, as in clinical practice ECGs are rarely interpreted without clinical details from the patient. Third, the cards and tests used ECG examples were carefully selected for having one clear abnormality. In clinical practice, a given ECG may have multiple abnormalities. Next, there was the potential in this study for priming bias to have impacted on students’ performances whereby participants may have been trained to look for the specific cardiac abnormalities targeted in this study. However, we assert that this is unlikely to have impacted upon the outcomes achieved as both intervention and control were made aware, and reminded of, the targeted cardiac conditions. Further, a substantial proportion of the SAFMEDS cards (21.4%) and the test images (14.3%) showed normal cardiac activity, which was emphasised to participants and which required participants to consider all images presented more fully. Nonetheless, future research evaluating SAFMEDS as an intervention may wish to consider how priming bias could be controlled for. Finally, participants volunteered to be involved in this study. It is possible that this resulted in a selection bias, whereby they were more highly motivated than the general student population and that this influenced the outcomes.

### Recommendations for future research and practice

First, SAFMEDS may rely in part on pattern recognition or non-analytic processing to build fluent performance, as opposed to deliberate analysis [34]. Rapid pattern recognition processes are often used by experts in a given field [34, 35] and several clinical guidelines emphasise the need for speed in addition to accuracy when interpreting ECGs to ensure optimal patient care [36, 37]. However, encouraging pattern recognition in the interpretation of ECGs could potentially encourage more surface learning than a deeper understanding of the physiology behind ECG abnormalities [35]. Therefore, medical educators implementing fluency teaching techniques should provide ample opportunities to practice the targeted behaviour across a variety of contexts with novel stimuli and situations of growing complexity [38] so that learners are encouraged to use pattern recognition in addition to, rather than instead of, analytical processes [34]. Further, the use of such varied and complex stimuli within SAFMEDS training is of interest as it may elucidate which stimuli (ECGs or other appropriate visual stimuli within different content areas) are most prone to error, take longest to learn, are least familiar to students, and so on. Such data would usefully inform the teaching within the specific content area and may reflect an additional use of applying the SAFMEDS methodology.

Second, there is a need for future SAFMEDS research to assess for evidence of retention (i.e., persistence of learning over time), and stability (i.e., persistence of learning during distraction) and generalisation, or transfer, of target behaviours post-intervention [1]. SAFMEDS research in other educational domains has evidenced retention and stability post-intervention [28]. Further, there is some medical education research to suggest that retention and stability are present after a procedural skill is taught to fluency, and that generalisation of learning to the clinical setting is observed [3].

Third, future research should examine the impact of the graphical (SCCs) and written feedback that was provided to participants within the current study. The SCC and associated feedback may be considered to constitute a ‘hidden’ component of the current intervention and it is not apparent what degree of impact of the intervention is attributable to this component alone. SCCs are widely used to record behaviour and improve performance [26]. The use of the SCC is sometimes presented as a core component of the SAFMEDS intervention [28], but has not been used in other studies of SAFMEDS in medical education [2, 22]. Researchers could usefully assess the impact of SAFMEDS with and without the provision of this feedback in order to empirically establish the degree of change which appears attributable to this detailed feedback alone. Alternatively, researchers could examine the contribution of the SCC within other medical education interventions within which a target behaviour can be defined and measured.

Finally, it has not been established how best to integrate fluency-based instructional methodologies into increasingly complex and congested medical curricula [39]. SAFMEDS may constitute a brief but powerful adjunct to usual teaching that, if made available, could be undertaken as “homework” independently by students. Careful consideration should be given to where SAFMEDS might best be employed. Further, while SAFMEDS may be suitable for improving performance of certain clinical skills (e.g., ECG interpretation, radiological imaging interpretation), other forms of fluency teaching, potentially using simulation-based technology [3] would be required for targeting procedural skills.

## Conclusion

Our study found that medical students trained in ECG interpretation using SAFMEDs for a relatively short duration showed significantly greater accuracy in electrocardiogram interpretation than students who received teaching as usual without SAFMEDS. Therefore, we suggest that SAFMEDS constitutes a learner-centric interventional strategy that may produce significant improvement in the performance of key clinical skills in a short amount of time. Further research must ascertain the place of SAFMEDS in medical education, appropriate target behaviours, and explore the retention, stability and generalisation of resultant learning.

## Data Availability

The datasets used and/or analysed during the current study are available from the corresponding author on reasonable request.

## Abbreviations

SAFMEDS: Say-All-Fast-Minute-Every-Day-Shuffled
ECG: Electrocardiogram
RCT: Randomised controlled trial
ANCOVA: Analysis of Covariance
SCC: Standard celeration chart
